# Notes

**Published:** 1901-03

**Authors:** 


### NOTES.

An outbreak of anthrax among a valuable lot of horses in New York State during the summer and autumn of 1900 caused the death of twelve, valued at a sum exceeding $35,000. Between November 21st and 27th 102 head received each two inoculations with anthrax vaccine virus, after which period no further loss ensued.

Baltimore is surely one of the greatest cities in the land for the development of veterinary fakirs. Whether the people of that city are merely credulous and easy, or have been used so long, both in high and low places, to empirical methods that they measure only the degree of fakirism to accept, is a question; but for cure-alls, food substitutes, and proprietary remedies it must be a city where nearly every man is his own horse-doctor.

A correspondent of the JOURNAL takes issue with a recent communication to the JOURNAL upon the efficacy of carbolic acid in tetanus, on the ground that this remedy has no action upon the tetanic poison, not destroying the spores in any strength used. He further comments upon the tendency of a certain percentage of cases to recover under quietude and freedom from excitement.

The work of weeding out tubercular cattle in New York State continues with the same safe results under the tuberculin test. A Delaware County herd recently tested found fifteen tubercular animals out of the twenty-three; these animals were destroyed and all found to be seriously diseased. A semi-public examination was held, and many who were disposed to be doubtful as to the value of tuberculin were more than convinced of its value as a diagnostic agent.

The Pan-American Exposition will hold some interesting competitive tests of the various breeds of cattle during the exhibition.

*Bulletin No.* 63 of the Arkansas Agricultural Experiment Station, by Dr. R. R. Dinwiddie, covers the research work done by the author in the field of tuberculosis. It treats of the "Relative Susceptibility of the Domestic Animals to the Contagia of Human and Bovine Tuberculosis."

It is estimated that there are about 400,000 Angora goats in the United States and that our annual production of mohair is about 1,000,000 pounds. Although very little has been said or written about Angora goats during the last forty years, they have been extensively bred in the Western States and Territories, especially in Texas, New Mexico, Nevada, Florida, California, and Oregon. Investigations prove that they are not only classed among the most useful of the domestic animals, and have been so classed for thousands of years, but their usefulness is manifested in various ways. The fleece, called "mohair," furnishes some of the finest fabrics among ladies' goods and is used in various other manufactures. Their habit of browsing enables the farmers in a wooded locality to use them to help in subjugating the forest. Their flesh is exceedingly delicate and nutritious; the milk, though not so abundant as with the milch breed of goats, is richer than cow's milk; their tanned skins, though inferior in quality to the skins of the common goat, are used for leather; their pelts make the neatest of rugs and robes, and they are excellent pets for children. A few of them in a flock of sheep are a protection from wolves and dogs, and their manure is noticeably helpful to the grass which follows them after they have cleaned away the underbrush. There is much interest in the goat question, and the United States Department of Agriculture has received numerous letters of inquiry concerning Angora goats. For the purpose of answering the many questions contained in these letters the Bureau of Animal Industry of that Department has just issued *Bulletin No.* 27, Bureau of Animal Industry, entitled "Information Concerning the Angora Goat." The bulletin was prepared by Mr. George Fayette Thompson, editorial clerk of the Bureau, and contains much information concerning the origin, history, and uses of this domestic animal. The bulletin is illustrated with a frontispiece and seventeen plates, and is for sale to miscellaneous applicants by the Superintendent of Documents, Union Building, Washington, D. C., at fifteen cents, the price affixed by him.

---

Dr. John Oliver, of Columbus, Miss., conducts a feed, sale and training stable in connection with his veterinary practice.

Dr. H. W. Witmer, of Shippensburg, Pa., has recently purchased a very centrally located plot of ground upon which he purposes erecting a very complete veterinary hospital.

Dr. Frank T. Shannon, of the Bureau of Animal Industry, has been transferred from Seattle, Washington, to Kansas City, Mo.

